# Optical Properties of Ferroelectric Epitaxial K_0.5_Na_0.5_NbO_3_ Films in Visible to Ultraviolet Range

**DOI:** 10.1371/journal.pone.0153261

**Published:** 2016-04-13

**Authors:** E. Chernova, O. Pacherova, T. Kocourek, M. Jelinek, A. Dejneka, M. Tyunina

**Affiliations:** 1 Institute of Physics of the Czech Academy of Sciences, Na Slovance 2, Prague 8, Czech Republic, 18221; 2 Czech Technical University, Technicka 2, Prague 6, Czech Republic, 166 27; 3 Microelectronics and Materials Physics Laboratories, University of Oulu, P. O. Box 4500, FI-90014 Oulun yliopisto, Finland; Gazi University, TURKEY

## Abstract

The complex index of refraction in the spectral range of 0.74 to 4.5 eV is studied by variable-angle spectroscopic ellipsometry in ferroelectric K_0.5_Na_0.5_NbO_3_ films. The 20-nm-thick cube-on-cube-type epitaxial films are grown on SrTiO_3_(001) and DyScO_3_(011) single-crystal substrates. The films are transparent and exhibit a significant difference between refractive indices *Δn* = 0.5 at photon energies below 3 eV. The energies of optical transitions are in the range of 3.15–4.30 eV and differ by 0.2–0.3 eV in these films. The observed behavior is discussed in terms of lattice strain and strain-induced ferroelectric polarization in epitaxial perovskite oxide films.

## Introduction

Perovskite-structure oxide ferroelectric crystals have long been used for optical applications due to their good nonlinear optical properties, high transparency and large index of refraction in the visible range [1, 2]. Advanced integrated optoelectronic and photonic applications can be implemented using thin single-crystal epitaxial films instead of bulk crystals [3–6]. However, epitaxial growth of ferroelectric films on dissimilar substrates is known to result in different phases of the crystal and electronic structures as well as ferroelectric polarization; such behavior is significantly different from those of bulk prototype crystals [7–10]. Correspondingly, the optical properties of epitaxial ferroelectric films can differ from those of the crystals [11, 12]. Knowledge of the epitaxial effects on the optical properties is vital for emerging thin-film ferroelectric applications.

Here, we experimentally study the optical properties of epitaxial ferroelectric K_0.5_Na_0.5_NbO_3_ (KNNO) films. Recent intensive research on bulk ceramic KNNO has been stimulated by the excellent piezoelectric properties of this solid solution, which is an environmentally friendly alternative to traditional lead-containing piezoelectrics [13]. In addition, potential optical applications of KNNO have been explored [14–17]. The room-temperature crystal structure of KNNO was identified as monoclinic [18]. By growing KNNO on top of a cubic substrate, it is possible to induce tetragonal crystal symmetry and lattice strain in the KNNO film. Here, such films are obtained using SrTiO_3_ and DyScO_3_ substrates. Our experiments reveal significant difference between the optical properties of these films. The observations are discussed in terms of lattice strain and strain-induced ferroelectric polarization in the films.

## Experiment

Epitaxial KNNO films with thickness of approximately 20 nm were grown via pulsed laser deposition on SrTiO_3_ (001) and DyScO_3_ (011) single-crystal substrates. The substrate temperature of 700°C and oxygen pressure of 20 Pa were maintained during deposition, and the oxygen pressure was 800 Pa during post-deposition cooling. The room-temperature crystal structure of the films was studied by x-ray diffraction (XRD) on a Bruker D8 DISCOVER SUPER SPEED SOLUTION diffractometer using the Cu-K*α* radiation. The lattice parameters were estimated from the positions of Bragg diffractions using EVA software and taking substrates as a reference.

**Fig 1:**
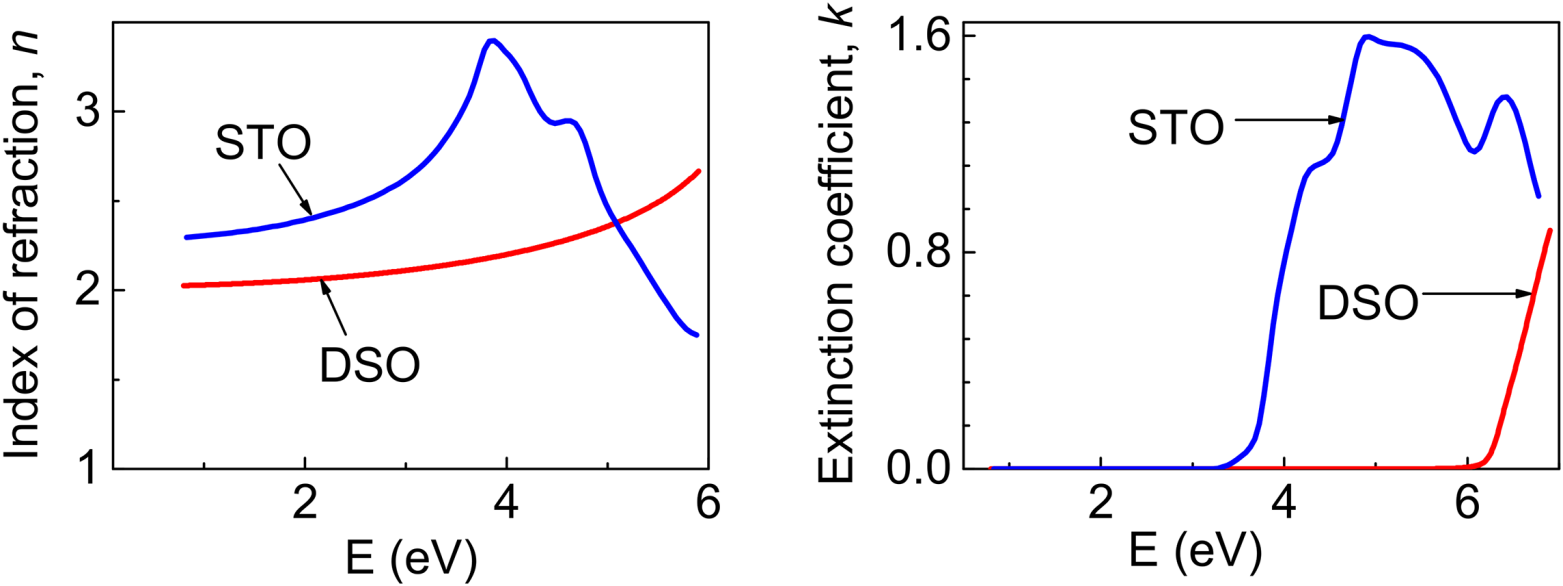
Index of refraction (a) and Extinction coefficient (b) of SrTiO_3_ and DyScO_3_ substrates.

The optical properties of the films were investigated by variable-angle spectroscopic ellipsometry on a VUV J. A. Woollam ellipsometer. Ellipsometric angles *ψ* and *δ* were measured at a photon energy range from 0.74 to 4.5 eV with a step of 0.02 eV. The measurements were performed in the reflection mode at multiple incidence angles in the range from 60° to 75° at room temperature. The obtained ellipsometric spectra were analyzed using a WVASE32 software package [19]. As a reference, the SrTiO_3_ and DyScO_3_ substrates were inspected independently under similar conditions. The optical constants of the substrates extracted using a model of semi-infinite substrate considering surface roughness, and ambient air are presented in Fig 1. The optical constants of the films were determined using a four-phase model that includes a semi-infinite substrate, one homogeneous layer, surface roughness, and ambient air. The optical constants of the substrate were kept as fixed parameters, and the thickness and oscillator parameters of the films were used as variables in the fitting procedure. The dielectric functions of the films were obtained using the Generalized Oscillator Model in the WVASE 32 software package [19, 20]. An analytical multi-oscillator model was first employed to determine the initial dielectric function and layer thicknesses. The obtained thicknesses of the films were in a good agreement with those determined by XRD analysis. To increase the accuracy of the data fitting procedure in weakly absorbing region [21] we additionally used the numerical inversion to extract optical constants over the full measured spectral range. This approach limits sensitivity to surface roughness as the absorbing region of the material at higher photon energies is ignored. Such fitting was done with the fixed thickness. The surface roughness was represented as a mixture of 50% solid and 50% voids, according Effective Medium Approximation [22]. The estimated thickness of the surface roughness layer was less than 2 nm for the KNNO thin-film samples.

## Results and Discussion

### Epitaxy

**Fig 2:**
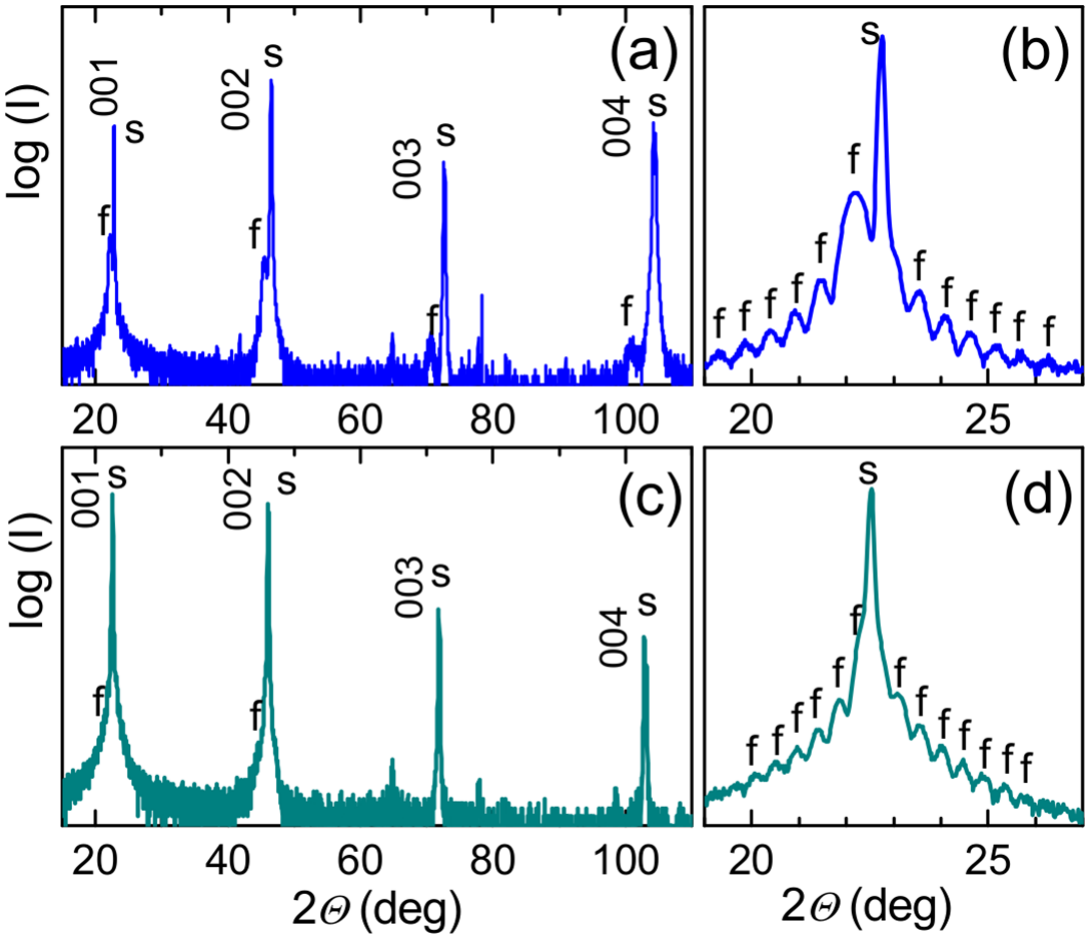
Θ-2Θ x-ray diffraction patterns in the KNNO films on (a,b) SrTiO_3_(001) and (c,d) DyScO_3_(011) substrates. Diffractions from the substrates and films are marked by *s* and *f*, correspondingly. Panels (b,d) show scans around (002) diffractions.

**Fig 3:**
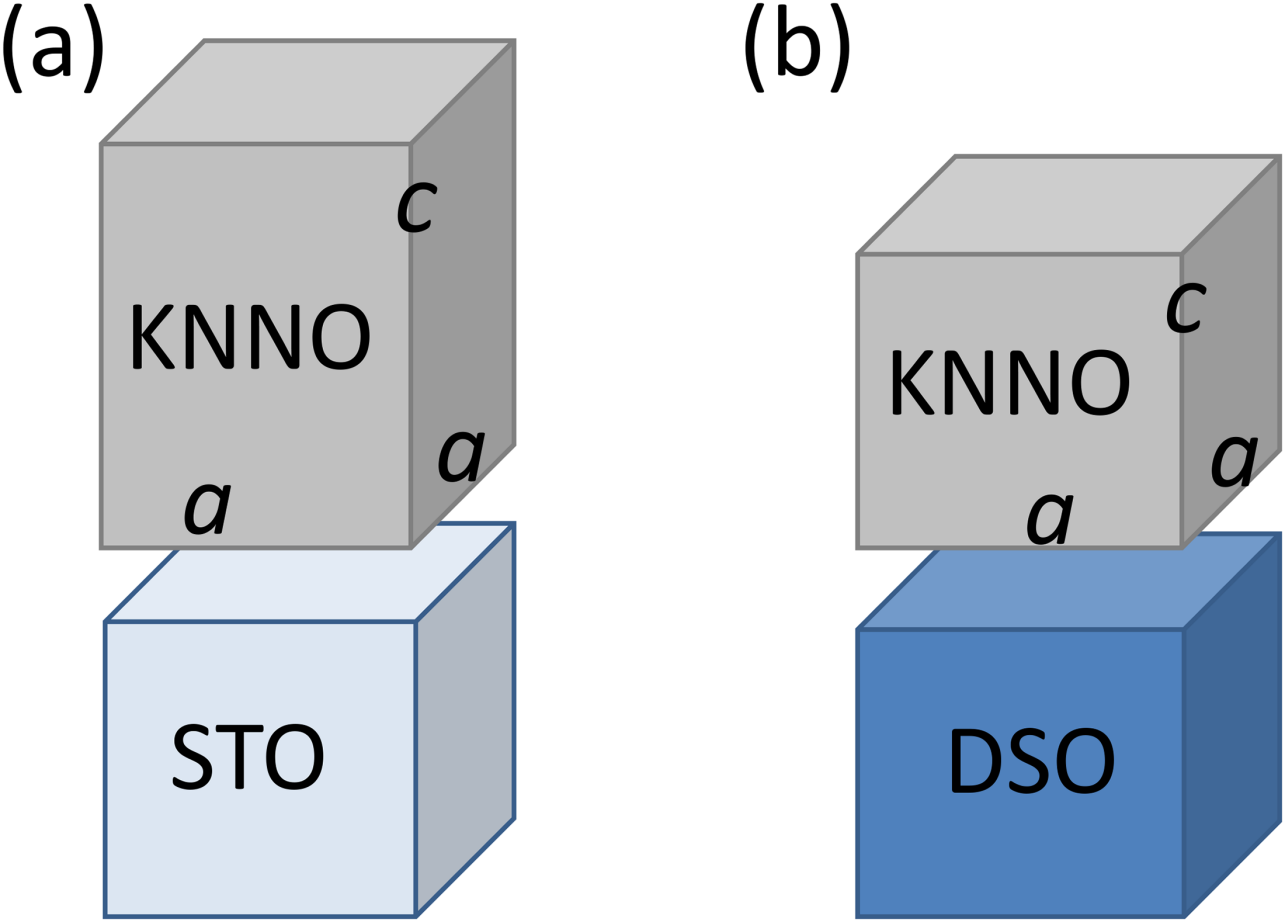
Schematic pesentation of (a) tetragonal and (b) pseudo-cubic unit cells in the cube-on-cube-type epitaxial KNNO films on (a) STO and (b) DSO substrates.

Cube-on-cube-type epitaxial growth of the KNNO films is revealed by XRD analysis. The Θ-2Θ scans show that the films are perovskite, highly oriented, with the (00*l*) planes parallel to the (001) planes of STO and (011) planes of DSO (Fig 2a–2d). The presence of Laue satellites indicates the high crystal quality of the KNNO films. The thickness of the films estimated from the Laue diffractions is approximately 20 nm. The reciprocal space maps inspected around (303) and (30$\bar{3}$) diffractions of STO and (336) and (33$\bar{6}$) diffractions of DSO evidence in-plane epitaxy with the [100] directions of KNNO being parallel to the [100] directions of STO and to the [110] directions of DSO. The out-of-plane and in-plane lattice parameters, *c* and *a*, respectively, are found to be *c* = 3.991Å and *a* = 3.931Å in the KNNO film on STO, and *c* = 3.968Å, *a* = 3.976Å in the KNNO film on DSO. The crystal structure of the KNNO film on STO can be interpreted as tetragonal with the longer out-of-plane lattice parameter (*c* > *a*) (Fig 3a). Compared to the monoclinic unit cell in the KNNO ceramics [18], the epitaxial KNNO films possess different crystal structures, lattice parameters, and unit cell volumes (Table 1). To estimate lattice strain in the films, the lattice parameter *a*_0_ of a prototype cubic KNNO cell is calculated as *a*_0_ = *V*^1/3^ = (*a*_*b*_ *b*_*b*_ *c*_*b*_)^1/3^, where *a*_*b*_, *b*_*b*_, and *c*_*b*_ are the lattice parameters of bulk KNNO. The films experience biaxial compressive in-plane strain *s* = *a*/*a*_0_−1, which is large (∼1.3%) in the KNNO/STO and close to zero (0.1%) in the KNNO/DSO. Considering theoretical phase diagrams of epitaxial KNbO_3_ and NaNbO_3_ films [8], the ferroelectric *c*-phase and *r*-phase can exist in the KNNO films on STO and DSO, respectively. The phases differ in the directions and magnitudes of ferroelectric polarization. Next, we show that this differences can lead to dramatic differences in the optical properties of the films.

**Table 1:** Summary of structural and optical properties in ceramics and thin-film KNNO.

	Ceramics [18]	KNNO on STO	KNNO on DSO
Structure	monoclinic	tetragonal	pseudo-cubic
*a*(*Å*)	4.0046	3.931	3.976
*b*(*Å*)	3.9446	3.931	3.976
*c*(*Å*)	4.002	3.991	3.968
*c*/*a*	—	1.015	0.998
*V*(*Å*^3^)	63.22	61.67	62.73
*n* (at 2 eV)	—	1.6	2.12
*E*_0_ (*α* = 10^4^ cm^−1^) (eV)	—	3.72	3.55
*E*_*i*_ (indirect) (eV)	—	3.53±0.02	3.15±0.02
*E*_*d*_ (direct) (eV)	—	4.08±0.02	4.30±0.02

### Optical properties

**Fig 4:**
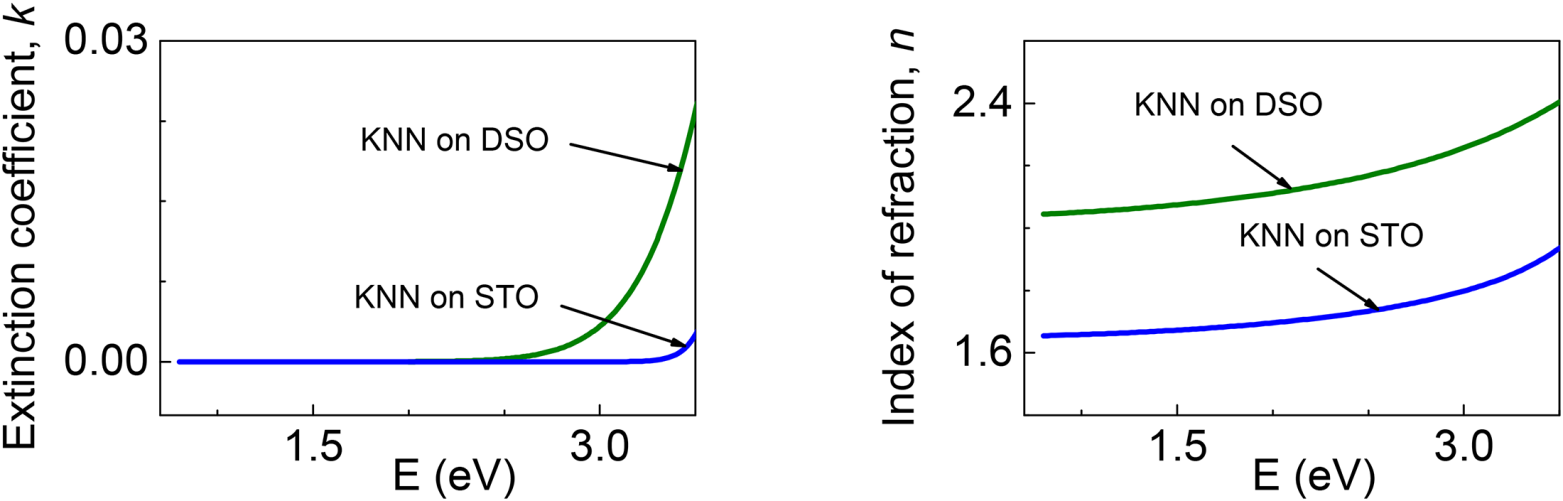
(a) Extinction coefficient *k* and (b) refractive index *n* of KNNO films.

The complex index of refraction n* = *n* + *ik* is determined in the KNNO films at room temperature. The extinction coefficient *k* is close to zero over a wide spectral range (Fig 4a). The results show that the films exhibit high transparency in the visible optical range, which is important for applications. The spectra of the refractive index *n*(*E*) in Fig 4b agree with those typically observed in the transparency range in perovskite oxide ferroelectrics [23]. In addition, the magnitude of *n* in the KNNO film on DSO is close to the typical values. However, the refractive index *n* is dramatically suppressed in the KNNO film on STO. The difference between refractive indices in the KNNO films is huge: the difference is approximatelyΔ*n* = 0.5. Note that KNNO belongs to a family of perovskite oxide ferroelectrics, where *d* orbitals of *A*-site cations contribute to electronic band structure (e.g., BaTiO_3_, but not PbTiO_3_). The refractive index is connected with electrical polarization through quadratic electro-optic effect in such materials [24]. A semi-empirical relationship is valid in the transparency range, far from edge of absorption:

$$\Delta \left(\frac{1}{n^{2}}\right)\approx gP^{2}$$ (1)

Here, the difference Δ(1/*n*^2^) is taken in relation to the paraelectric state with zero polarization, *P* is the polarization in the ferroelectric state, and *g* is the quadratic electro-optic coefficient. The tensor nature of the coefficient *g* is ignored in expression (1) for simplicity. The coefficient *n* decreases with increasing spontaneous ferroelectric and/or electric-field-induced polarization. The observed huge difference between the coefficients *n* in the KNNO films can be caused by different polarization therein. As is known, a strong coupling between lattice strain and polarization is the characteristic feature of perovskite oxide ferroelectrics. Compared to the KNNO film on DSO, the larger lattice strain causes larger polarization, which, in turn, leads to smaller index of refraction *n* in the KNNO film on STO.

**Fig 5:**
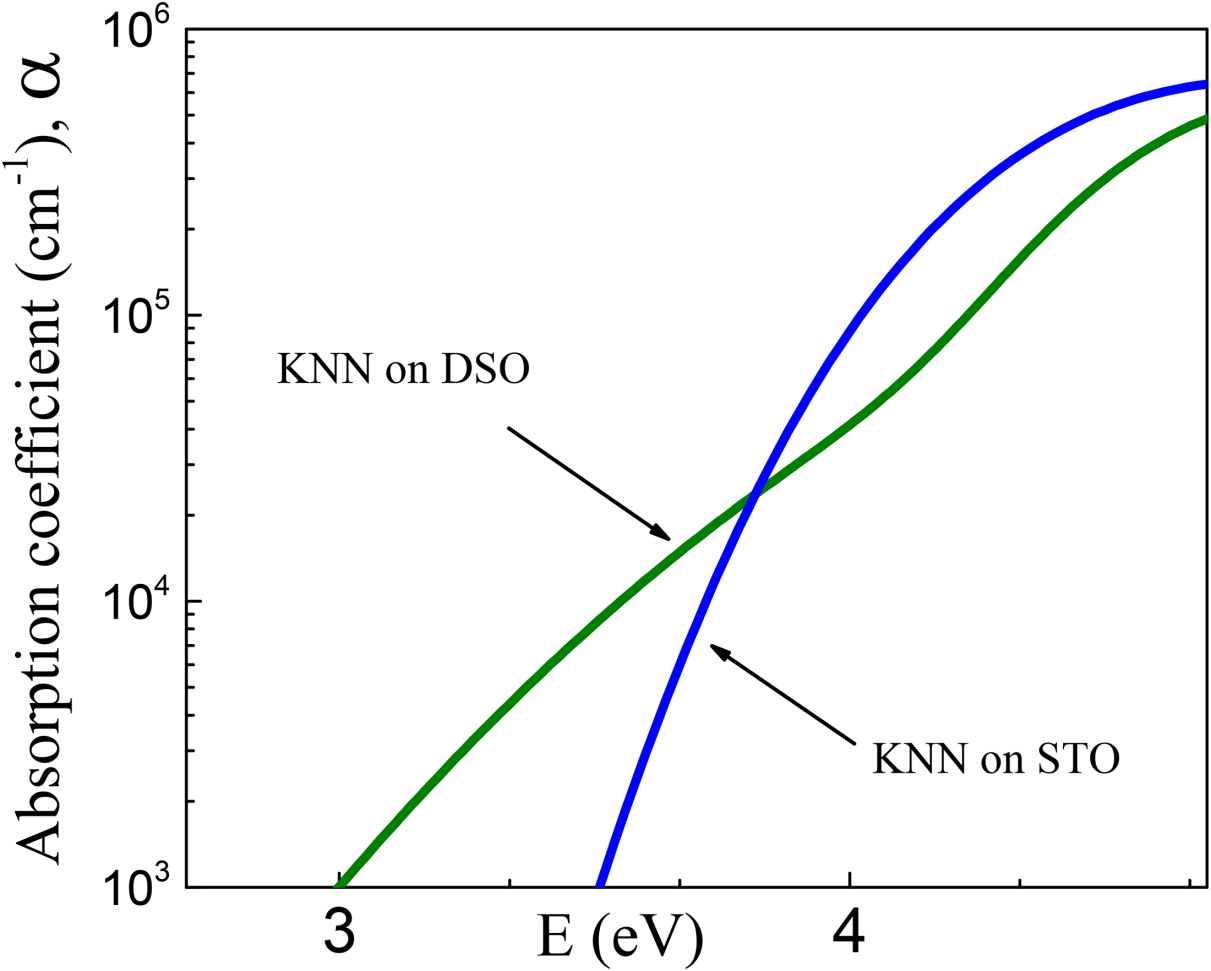
Absorption coefficient *α* as a function of photon energy *E* in the KNNO films.

**Fig 6:**
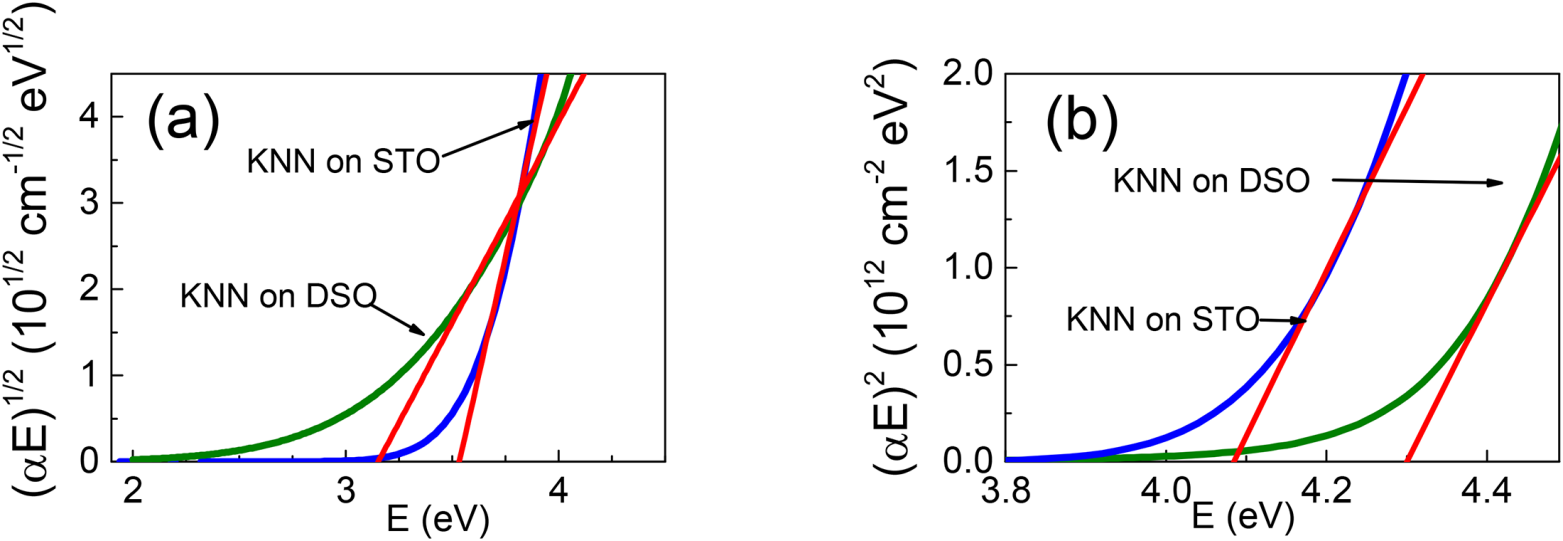
Tauc plots for (a) indirect and (b) direct optical transitions in the KNNO films.

The microscopic mechanism behind the expression (1) is connected with an increase of electron band energies, especially of valence-band energies, which is induced by polarization in ferroelectrics [23]. The increase of valence-band energy can manifest itself in a widening of the band gap and an increase of the energies of interband optical transitions. Correspondingly, shifts of absorption edge and other spectral features to higher photon energies, i.e., blue shifts, are expected with increasing ferroelectric polarization. To detect such shifts and thus verify the suggested influence of strain-induced ferroelectric polarization on index of refraction in the KNNO film, the spectra of optical absorption *α*(*E*) are analyzed (Figs 5 and 6).

Often, the optical band gap is determined from Urbach tail [25], where *α*(*E*) decreases exponentially with decreasing *E* below energy gap [26]:

$$\alpha \left(E\right)=\alpha_{0}exp\left[\frac{\left(E-E_{t}\right)}{E_{u}}\right]$$ (2)

Here, *E*_*u*_ describes the slope of absorption tail, *E*_*t*_ is the energy, below which the Urbach behavior is observed, and *E*_*g*_ is the band-gap energy. As seen from Fig 4, the logarithmic plots of absorption coefficient *α* cannot be fit by a linear function corresponding to the expression (2). The absorption in the KNNO films is free of Urbach tails. Simultaneously, one can notice a clear difference between the spectra in the two films.

To quantify this difference, we use the energy *E_α_*, at which absorption coefficient is *α* = 10^4^ cm^−1^. The energy is *E_α_* = 3.55 eV in the film on DSO, and it is larger, *E_α_* = 3.72 eV, in the film on STO. The observed blue-shift of absorption edge in the film on STO with respect to that in the film on DSO is consistent with the above-discussed effect of strain-induced polarization. The effect of polarization is further verified by comparing energies of optical transitions in the films.

The energies of transitions are determined using the widely employed Tauc relations [27]. The Tauc plots for both indirect and direct transitions are analyzed. The analysis reveals indirect bandgapin the films (Fig 5a). The bandgap energy *E*_*i*_ is obtained from the linear fits to [(*αE*)^1/2^ ∝ *E*] in the range of *α* = (2-45)×10^3^ cm^−1^. Again, the bandgap energy *E*_*i*_ = 3.53 eV in the film on STO is clearly larger than the bandgap energy *E*_*i*_ = 3.15 eV in the film on DSO. This result is consistent with the suggested effect of strain-induced polarization.

In addition, good linear fits to [(*αE*)^2^ ∝ *E*] for *α* = (1-3)×10^5^ cm^−1^ (Fig 6b) evidence the presence of a direct optical transition with the energy *E*_*d*_ in both of the KNNO films. The energy is *E*_*d*_ = 4.08 eV in the film on STO, which is smaller than the energy *E*_*d*_ = 4.30 eV in the film on DSO. Thus, in contrast to the polarization-caused blue shift of the indirect transition, the higher-energy direct transition is found to red-shift with increasing lattice strain (and, hence, with strain-induced polarization) in KNNO. The observed controversial behavior of optical transitions suggests more complex changes in the band structure. In particular, besides the suggested polarization-induced increase of valence-band energies, band splitting can be caused by ferroelectric polarization [28]. Such splitting may be responsible for the apparent red-shift of *E*_*d*_.

Theoretical analysis of optical properties in strained epitaxial ferroelectric films is highly desirable to understand such large variations in behavior as those observed in the present and other works [29–31]. Nevertheless, despite the lack of theoretical support, our experimental results strongly suggest that optical refraction and absorption can be tuned using epitaxial growth of ferroelectric films. The epitaxial-deposition induced changes of optical properties can be employed in creating materials for advanced optoelectronic and photonic applications.

## Conclusion

The complex index of refraction in the spectral range of 0.74 to 4.5 eV is studied in 20-nm-thick cube-on-cube-type epitaxial films of ferroelectric K_0.5_Na_0.5_NbO_3_. The films are grown on SrTiO_3_ (001) and DyScO_3_ (011) single-crystal substrates, and experience biaxial in-plane compressive strain. Compared to the weakly strained film on DyScO_3_ (011), the strongly strained film on SrTiO_3_ (001) exhibits a smaller refractive index in the transparency range and a larger bandgap energy. The observed behavior is explained using a semi-empirical model of the electro-optic effect in ferroelectrics and considering strain-induced polarization in epitaxial films. The results are important for the development of materials for future optoelectronics and photonics.
